# Assessment of DNA vaccine encoding *Toxoplasma gondii* microneme complete gene and IL-12 as adjuvant in BALB/c mice

**DOI:** 10.22038/ijbms.2019.34872.8276

**Published:** 2019-08

**Authors:** Fatemeh Ghaffarifar, Mohammad Jafarimodrek, Hossein Vazini, Zohreh Sharifi, Abdolhossein Dalimi, Mohammad Saaid Dayer

**Affiliations:** 1 Department of Parasitology and Entomology, Faculty of Medical Sciences, Tarbiat Modares University, Tehran, Iran; 2 Department of Medical Parasitology, Faculty of Medicine, Zahedan University of Medical Sciences, Zahedan, Iran; 3 Nursing Department, Basic Sciences faculty, Hamedan Branch, Islamic Azad University, Hamedan, Iran; 4 Blood Transfusion Research Center, High Institute for Research and Education in Transfusion Medicine, Tehran, Iran

**Keywords:** BALB/c mice, DNA vaccine, Immunization, pCAGGS-IL12, pc-MIC3, Toxoplasma gondii

## Abstract

**Objective(s):**

*Toxoplasma gondii* is an obligate intracellular protozoan parasite that causes toxoplasmosis in humans and animals. Micronemes (MICs) are effective candidates for DNA vaccine.

**Materials and Methods:**

In this study, we evaluated the immune response of BALB/c mice against *MIC3* gene of *Toxoplasma gondii* and interleukin 12 (IL-12) as DNA vaccine. The *MIC3* gene was cloned into the PTZ57R/T vector before sub-cloning in pcDNA3. Recombinant pc-MIC3 was transformed into *Escherichia coli* (TOP10 strain). The pc-MIC3 plasmid was then transfected into Chinese Hamster Ovary (CHO) cells, and the expression of the *MIC3* gene was evaluated by SDS-PAGE and Western blotting. Sixty female BALB/c mice were divided into 6 groups. Each group received 3 intramuscular immunizations on days 0, 21^st^ and 42^nd^ using one of the following stimulants: phosphate-buffered saline, pcDNA3, pCAGGS-IL12, pc-MIC3 (100 µg), pc-MIC3 (50 µg), or combined pCAGGS-IL12 (50 µg) and pc-MIC3 (50 µg). The enzyme-linked immunosorbent assays was applied to evaluate interferon gamma (IFN-γ) and IL-4 cytokines excretion of lymphocytes stimulated with tachyzoites lysate antigen, as well as the total levels of immunoglobulin G (IgG), IgG2a and IgG1 in immunized mice sera.

**Results:**

Our results showed that mice challenged with pc-MIC3 (100 µg) had the highest longevity and quantity of immunoglobulin. Moreover, the highest expression level of IFN-γ was found in mice injected with combined pcMIC3 and pCAGGS-IL12 (*P<*0.05).

**Conclusion:**

The *MIC3* gene can be an efficient DNA vaccine candidate against toxoplasmosis. While, the single-gene vaccine can confer partial protection to mice against toxoplasmosis, the multigene vaccine can significantly enhance immune responses.

## Introduction

Toxoplasmosis is one of the most common parasitic infections caused by protozoa in the world. The causative agent of this disease is an obligate intracellular protozoan called *Toxoplasma gondii* that belongs to the phylum of Apicomplexa (1). This parasite infects not only humans but also a large number of mammals, as well as different types of birds. It can replicate in host cell in three forms of tachyzoites, bradyzoites (tissue cyst) and sporozoites (oocysts) (2). The parasite can cause severe neurological and eye complications in immunocompromised patients, whereas in infants born to infected mothers may result in hydrocephalus, microcephaly, calcification, or chorioretinitis (3-5). Given its widespread distribution, easy transmission and irreparable complications, toxoplasmosis needs to be prevented by anti-parasitic drugs, or vaccination (6). Today, immunization by plasmids containing DNA sequences encoding favorable antigens has opened a new horizon in vaccine production (7, 8). Some antigens of *T*. *gondii* including rhoptry proteins (ROPs), surface antigen glycoproteins (SAGs), excretory-secretory dense granule proteins (GRAs) and microneme proteins (MICs) are relatively effective candidates for DNA vaccine (9-11). The production of MIC proteins in microneme organelles such as prerequisite proteins for promotion of adhesion and invasion by *T. gondii* makes them suitable antigens for DNA vaccine production. The MIC3 protein plays an important role in identification of *T. gondii* and attachment to the host cells (12). Different studies showed that MIC3 is very immunogenic besides being encoded by a single-gene lacking introns (13). It is known as the main *T. gondii* antigen, which stimulates immunity response, hence attracting more immunological investigations (14-15). In the present study, *MIC3* gene was cloned and expressed in Chinese Hamster Ovary (CHO) cells. Also, immunization with a DNA plasmid encoding *T. gondii *MIC3 was assessed in BALB/c mice.

## Materials and Methods

***Ethics statement***

This project was approved by the Medical Ethics Committee of Tarbiat Modares University, Tehran, Iran, adopting the Declaration of Helsinki (1975) and Guidelines of the Society for Neuroscience concerning Animal Care and Use (1998).

***Animals and parasite***

Female BALB/c mice aged 8 weeks were bought from Razi Vaccine and Serum Research Institute (RVSRI). In this study, we used the tachyzoites (RH strain) of *T. gondii* that was stored at -80 ^°^C in the Parasitology Department of Tarbiat Modares University, Tehran, Iran.

***Gene amplification of MIC3***

DNA from parasite samples was extracted by phenol-chloroform and quantified by spectrophotometer using A260/280 ratio (16). The sequence of *MIC3* gene was obtained from the gene bank (www.ncbi.nlm.nih.gov) with the accession number AJ132530. Both reverse and forward primers were designed using the Gene Runner software (Table 1).

The PCR reaction was performed in a volume of 25 μl. *MIC3* gene was amplified by PCR during 35 cycles according to the following program: pre-denaturation at 94 ^°^C for 5 minutes, followed by 35 cycles at 94 ^°^C for 1 min, 56 ^°^C for 30 sec and 72 ^°^C for 50 sec, and final extension for 7 minutes at 72 ^°^C. The PCR product was analyzed by electrophoresis on 1% agarose gel. DNA was purified from the agarose gel with the aid of Agarose Gel DNA Extraction Kit (Bioneer, Germany) as per Sambrook *et al.* 2001 (17).

***Cloning of MIC3 gene and construction of recombinant plasmids***

*MIC3* gene was cloned in pTZ57R/T vector by InsTAclone^TM ^PCR cloning Kit (Fermentas, Lithuania). Bacteria (*Escherichia coli* TG1 strain) were prepared from culture containing calcium chloride before being transformed by the thermal shock in the presence of pT-MIC3. Bacteria were then cultured in LB (Luria Bertani) broth containing ampicillin antibiotic, X-gal, and isopropyl β-D-1-thiogalactopyranoside (IPTG). Plates were incubated at 37 ^°^C (16-18 hr. overnight). The plates were then placed at 4 ^°^C for either blue- (lacking pT-MIC3) or white-colony formation (containing pT-MIC3). The white colonies were collected from the cultures and the gene cloning was confirmed via PCR and sequencing methods (17).

**Figure 1:**
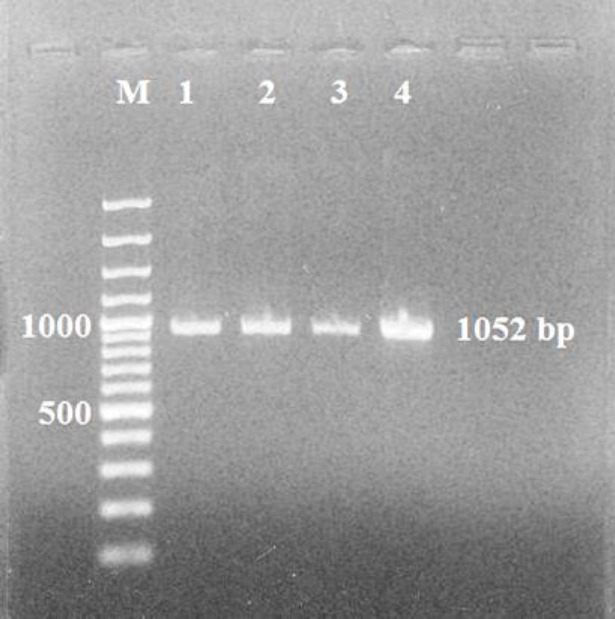
Electrophoresis of microneme 3 (MIC3) PCR product (1052 bp) equals to the size of toxoplasma *MIC3* gene in comparison with 100 bp DNA ladder on agarose gel

**Figure 2:**
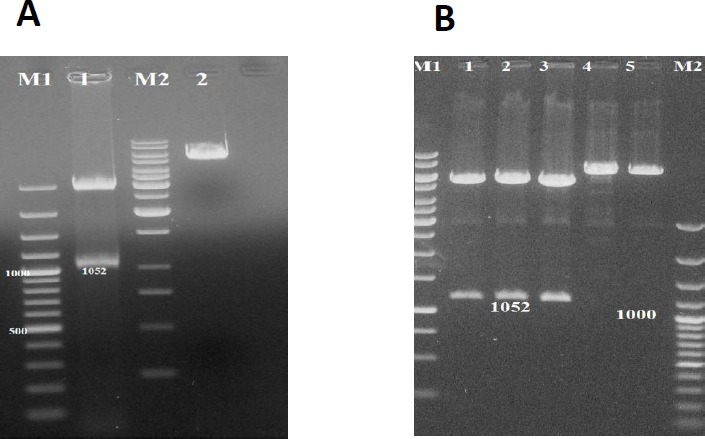
A: Electrophoresis of M1, 100 bp DNA ladder; Line 1, pT-MIC3 with double digestion with EcoRV and HindIII enzymes; M2, 250 bp DNA ladder; Line 2, pT-MIC3 with one digestion on agarose gel. B: Double digestion of pc-MIC3 (Lines 1-3) with EcoRV and HindIII enzymes and one digestion of pc-MIC3 (Lines 4-5) with EcoRV or HindIII enzymes, M1, 100 bp DNA ladder; M2, 250 bp DNA ladder

**Figure 3:**
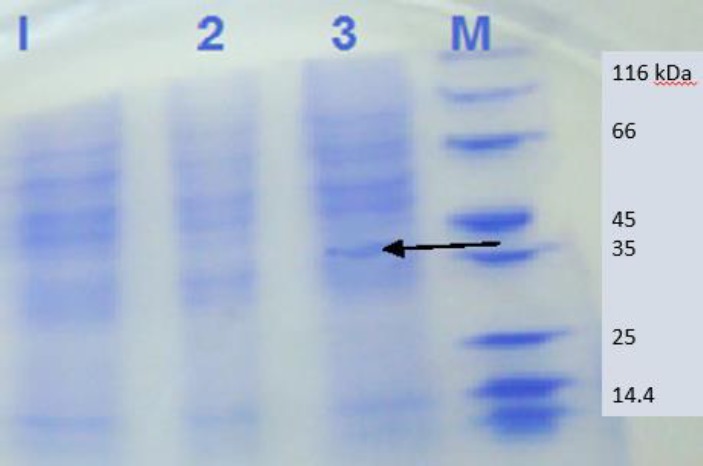
SDS-PAGE of transfected Chinese Hamster Ovary (CHO) cells with pc-MIC3 (line 3), pc-DNA (line 2) and empty CHO cells (line 1) in comparison with protein ladder (M)

**Figure 4:**
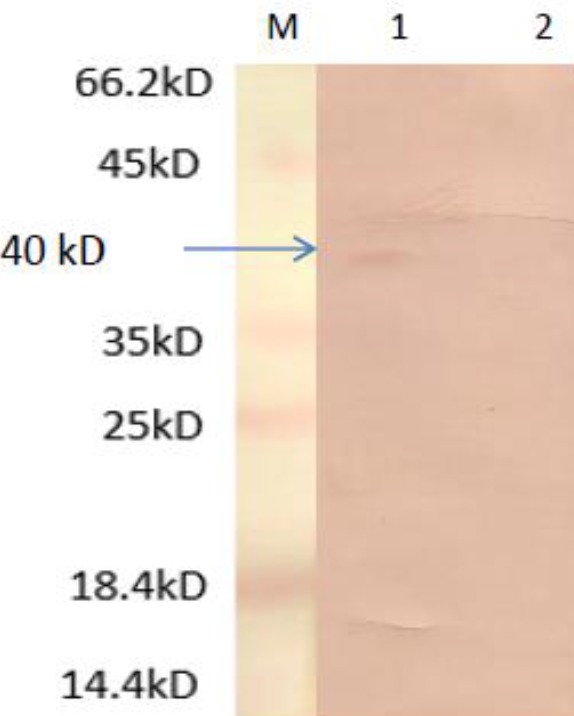
The result of Western blotting of transfected Chinese Hamster Ovary (CHO) cells with pc-MIC3 (line 1), and empty CHO cells (line 2) in comparison with protein ladder (M)

**Figure 5:**
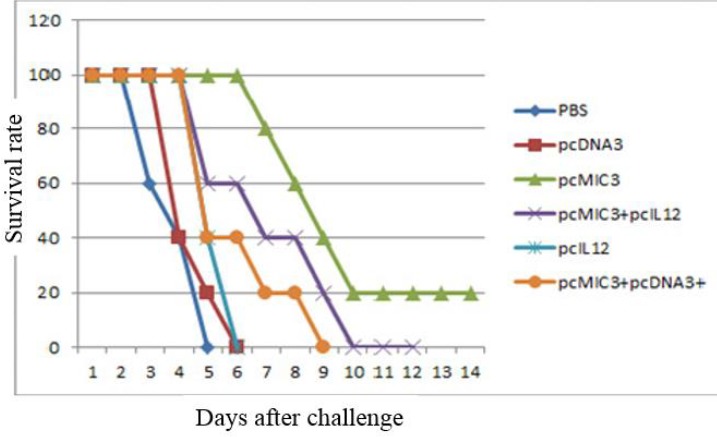
The survival rate of vaccinated BALB/c mice after challenge with 104 live *Toxoplasma gondii* (RH strain) tachyzoites. There is significant differences for pcMIC3 (100 µg) and pcMIC3+pcIL12 with control groups by Kaplan–Meier test (*P<*0.05)

**Figure 6:**
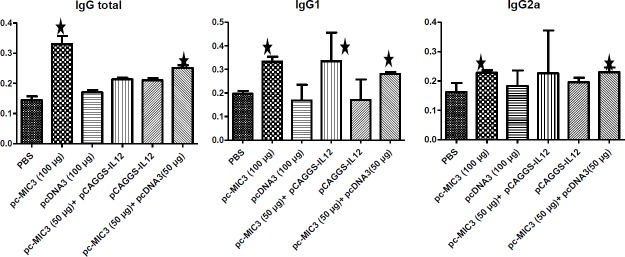
Mean±SD the optical density (OD) of total immunoglobulin G (IgG) and IgG1, IgG2a in mice sera of immunized and control groups

* There is significant differences with control groups (*P<*0.05)

**Figure 7:**
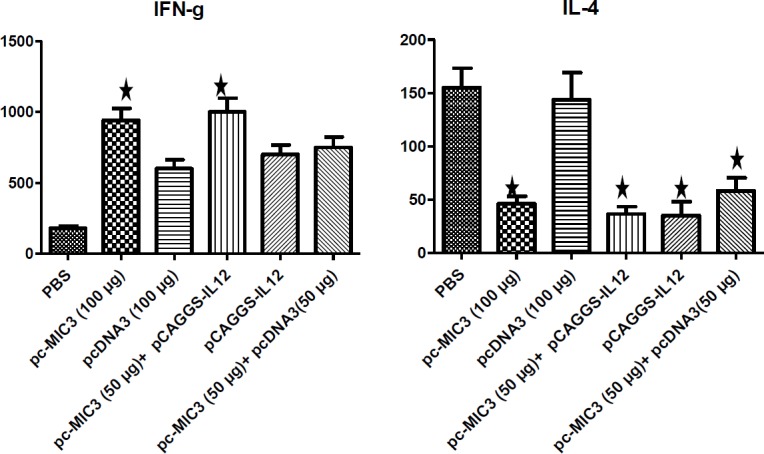
Mean ±SD of interleukin 4 (IL-4) and interferon gamma (IFN-γ) amount after culture of lymphocyte in 72 hrs in immunized mice of experimental and controlled group.* There is significant differences with control groups (*P<*0.05)

**Table 1:** Designing reverse and forward primers of *microneme* (*MIC*) gene with HindIII and EcoRV sequences that were added to reverse and forward primers, respectively

**Primer **	**The restriction enzymes site that added to primers **	**Nucleotide No**	**Sequence of Primers**
Forward	HindIII	31 nucleotides	5΄- CACA ↓ A GCTTATGGCGCTCACCTTCATGGGGG - 3΄
Reverse	EcoRV	32 nucleotide	5΄- ACAGAT ↓ ATCTCACGTCACGGTGTGGGCATGGT - 3΄

***Sub-cloning of MIC3 gene***

Two plasmids of pT-MIC3 and pcDNA3 were digested with HindIII and EcoRV enzymes, and the products were analyzed by electrophoresis on 1% agarose gel. The *MIC3* gene and pcDNA3 plasmid were then purified from agarose gel using a DNA extraction kit. The *MIC3* gene was ligated to pcDNA3 plasmid with aid of T_4_DNA ligase enzyme. The resulting pcMIC3 was transformed into the *E. coli* (TG1 strain) using a thermal shock. The bacteria were then cultured in a tube containing LB liquid and ampicillin at 37^ °^C (16-18 hrs overnight). To confirm gene cloning, enzymatic digestion process was performed using EcoRV and Hind III enzymes (17).

***Expression of pc-MIC3 in CHO cells***

For this experiment, 1-3×10^6^ CHO cells were cultured in Dulbecco’s Modified Eagle Medium (DMEM) containing fetal calf serum (FCS) 10% and 100 unit/ml of penicillin in addition to 100 mg/ml of streptomycin and incubated overnight in 5% CO_2 _at 37 ^°^C. The Fugen 6 Transfection Reagent Kit (Roche, USA) was used to transfect pc-MIC3 to the CHO cells. The product of *MIC3* gene was extracted from CHO cells by sonication and freeze-thaw technique. The density of MIC3 product was measured by Bradford method (18). 

***SDS-PAGE and Western blotting***

The molecular weight of the protein was evaluated through SDS-PAGE (19, 20). For Western blotting, the constructed protein bands were transferred to nitrocellulose paper and recognized by sera positive against toxoplasmosis, goat Anti-Human immunoglobulin G (IgG) antibody, horseradish peroxidase (HRP) conjugate (Sigma-Aldrich) and 3,3′-Diaminobenzidine (DAB) (Sigma-Aldrich) as the substrate (20).

***BALB/c mice immunization and challenge***

Immunization was assessed in sixty BALB/c mice, which had been divided into 6 groups (10 mice per each group). Three groups were allocated to control treatments, each received one intramuscular injection of phosphate-buffered saline (PBS; 100 μl), pcDNA3 (100 μg) or pCAGGS-IL12 (100 μg) in the quadriceps femoris muscle. Each of the three main experimental groups was administered with one injection of pc-MIC3 plasmid (100 μg), combined pcMIC3 plasmid (50 μg) and pCAGGS-IL12 plasmid (50 μg) or combined pc-MIC3 plasmid (50 μg) and pcDNA3 plasmid (50 μg). All mice received the same volume for injection that was equal to 100 μl. The immunization was undertaken at 3-week intervals on days 0, 21, and 42. Three weeks after the last immunization on day 63, mice were challenged with 1×10^4 ^tachyzoites (RH strain) recovered from peritoneal fluid. The experimental mice were then monitored daily to determine the fatality rates of different groups based on their protection against the disease (21).

***Humoral immunity***

From each group, 5 mice were randomly selected and bled from eyes on days 21, 42, and 63. The mice sera were collected and examined against toxoplasmosis by ELISA technique. The concentration of parasite antigen tachyzoites lysate antigen (TLA) was 20 µg/ml, and sera dilution 1:10 were used for ELISA. Also, subtypes of IgG antibody were measured by Monoclonal Antibody Isotyping Reagents Kit, and Capture ELISA method (22, 23).

***Cellular immunity***

Seven weeks after the third immunization, half of mice in each group were sacrificed and their spleens were scooped to prepare lymphocytes. The spleen lymphocytes were cultured in RPMI with 10% fetal bovine serum (FBS). The cells viability was tested by trypan blue. The lymphocytes (2×10^6^/ml) were incubated with TLA (20 µg/ml) for 72 hrs in 5% CO_2 _at 37 °C. The supernatant of cell cultures were then collected for cytokine assay. For evaluation of interleukin 4 (IL-4) and interferon gamma (IFN-γ) of cytokines, the supernatant was investigated by ELISA kit (Ucytech, Netherland). The standard curve was created for each kit, and the measurements of both IL-4 and IFN-γ cytokines were carried out according to the manufacturer’s instruction (23).

***Statistical analysis***

Statistical tests of Kruskal-Wallis, Kaplan Meiere, and Mann-whitne were applied to analyze the data using SPSS version 19. The results were reported as mean±standard deviation for each group. In all statistical analyses, the difference was considered significant at *P<*0.05 (16, 17).

## Results

***PCR amplification of MIC3*** ***and sequence determination***

Figure 1 shows that PCR amplified DNA has about 1052 bp, equals to the size of toxoplasma *MIC3* gene. Also, no gene has been amplified except MIC3. Therefore, the primers were found to be designed specifically for *MIC3* gene amplification.

The sequence analysis of *MIC3* gene of *T. gondii* in pTZ57R/T plasmid showed 100%, 99% and 98% similarity with Genbank-registered *MIC3* gene of *T. gondii* (RH Strain) with the accession numbers of JF330835.1, DQ676961.1 and AJ132530.1, respectively. 

***Construction of pcDNA-MIC3 plasmid***

Figure 2 shows enzymatic digestion of pTMIC3 and pcDNA3 by EcoRV and HindIII enzymes. After separation of *MIC3* gene from pT-MIC3, the MIC3 *gene* was sub-cloned in pcDNA3 plasmid. On agarose gel, the bands of pc-MIC3 plasmid were above those of pcDNA3 plasmid. This indicates that pcMIC3 plasmid is heavier than pcDNA3 plasmid; hence, the cloning of MIC3 portion of pcDNA3 plasmid was ensured. The formation of two bands of 5.4 kbp (equal to pcDNA3 weight without MIC3) and 1052 bp (equal to MIC3 weight) showed that this plasmid was cut into two portions by EcoRV and HindIII enzymes.

***Gene expression in CHO cells***

SDS-PAGE (Figure 3) showed that the loaded proteins were separated based on their molecular weights within the range of 14 to 116 kDa. The formation of protein band with molecular weight of about 40 kDa on nitrocellulose paper showed that MIC3 protein may be identified by Western blotting (Figure 4) with human anti-Toxoplasma IgG positive sera (24).

***BALB/c mice immunization and challenge***

The results showed that the three control groups treated with pcDNA3, PBS or pCAGGS-IL12 experienced mortality from day 4 post-challenge until day 6. All mice succumbed to death by day 6. However, for pcDNA3 and pcIL12 groups the survival rates were 40% at the end of 4^th^ day and zero at the end of 5^th^ day. In vaccinated experimental groups, the mortality of mice began from day 6 post-challenge for those immunized with pcMIC3/pCAGGS-IL12 and pcMIC3/pcDNA3, and from day 7 for pcMIC3 group. The pcMIC3/pcDNA3-challenged mice remained alive until the end of day 8, though the pcMIC3+pCAGGS-IL12- challenged mice survived until day 10. In term of survival rates, there were significant differences between those immunized with pcMIC3 (100 µg) and pcMIC3+pcIL12 and control groups (*P<0.05*) (Figure 5).

***Humoral immunity***

*The measurement of IgG*

Among the tested group, the PBS and pcMIC3 groups expressed very low and very high levels of IgG both in the first and second blood sampling, respectively. Significant differences in IgG levels (*P* ≤0.05) were also observed between the first and second blood sampling of each group (Figure 6).

***The measurement of IgG subtypes (IgG***_1_ ***, IgG***_2a_ ***)***

The lowest and highest level of IgG1 was recorded in pcMIC3/pCAGGS-IL12, and pcDNA3 groups, respectively. However, the titers of IgG_2a_ were higher in pcMIC3/pCAGGS-IL12 group and lower in PBS group (Figure 6).

***Cellular immunity***

*The measurement of IFN-γ and IL-4 cytokines*

After 72 hrs of lymphocyte culture, the highest and lowest amounts of IL-4 cytokine were found in PBS and pcMIC3/pCAGGS-IL12 groups, respectively. Whereas, the highest and lowest levels of IFN-γ cytokine were recorded in pcMIC3/pCAGGS-IL12 and PBS groups, respectively (Figure 7).

## Discussion

DNA vaccination is a powerful method for induction of cellular and humoral immune responses. Upon injection of DNA plasmid into the host, the host cells express the encoded protein (23). In general, it should be mentioned that DNA vaccination induces type 1 T helper (Th1) immune responses more than those of Th2 (24, 25). The vaccination efficiency depends more on the way of producing immunity. DNA vaccines are highly immunogenic and induce different immunity responses compared to normal ones (26). In these vaccines, DNA itself acts as an adjuvant. The existence of special sequence of DNA, enriched with non-methylated CpG motif in vector or added to vaccine formula, will intensify the response of Th1 pattern, and induce the actions of IL-12, IL-6 and IFN-γ (27). Previous studies about prophylaxis of toxoplasmosis disease showed that vaccination with excretory-secretory antigens of *T. gondii* like ROPs, GRAs, and MICs can create multifaceted immune responses against the parasite (10, 11). In this study, the immunity production of a plasmid encoding *T. gondii* MIC3 in BALB/c mice was investigated. The plasmid encoding MIC3 with or without adjuvant of IL-12 was injected in BALB/c mice. This study showed that IL-12 adjuvant improved survival rates of mice. 

The pc-MIC3 plasmid showed the ability to prolong the survival of vaccinated mice either with or without adjuvant, although it worked better when injected at 100 µg rather than at 50 µg, even if combined with IL-12 adjuvant. The level of immune response stimulation by an adjuvant depends on its composition and the way of its formulation with MIC3. Xue and his colleagues examined a multi antigen DNA vaccine expressing ROP2, and SAG1 against toxoplasmosis along with subunit of cholera toxin and IL-12 as the genetic adjuvant in BALB/c mice. They showed that multi antigen vaccine combined with IL-12 adjuvant elicited higher humoral and cellular (Th1) responses in mice compared to the stand-alone multi antigen vaccine (8). Xue evaluated a DNA vaccine containing SAG1, ROP2, and GRA2 of *Toxoplasma* in mice and reported higher Th1 responses by a plasmid containing 3 genes compared to that containing 2 genes. Based on the properties of genetic adjuvants (IL-12), we expected that the level of antibody in groups challenged with pc-MIC3 plasmid plus adjuvant will be higher than those received pc-MIC3 plasmid alone. However, we observed no increase of antibody in mice injected with pc-MIC3 plasmid along with IL-12 both at first and second phases of blood sampling. Desolmi stated that immunization of mice with concurrent administration of IL-12 and GRA4 plasmids resulted in an antagonistic effect and therefore decreased mice survival (28). Furthermore, the concurrent use of the plasmid and IL-12 as an adjuvant caused increase of immunity responses and survival in mice (29). Assessing a multi antigen vaccine, Xue and his colleagues showed that the vaccine had reduced efficacy unless its main genes were combined in fused form and applied with adjuvant (29). The results of the present study revealed that the pc-MIC3 plasmid can solely activate the immunity system and induce production of appropriative antibody. Addition of genetic adjuvant to a fusion form may have even more effects. Investigations of IL4 and IFN**-γ **cytokines showed that the pc-MIC3 plasmid can stimulate cellular immunity. The high production of IFN-γ activates macrophages and hinders the growth of parasites by demolishing tryptophan and actuating inducible nitric oxide synthase (iNOS) mechanism. Therefore, lymphokines and macrophages play important roles in immunity response of Th1 (30, 31). The reduced amount of IL4 in this study provided evidence in this connection. In terms of production of high quantity of IFN-γ and low quantity of IL-4, our findings were in conformity with Xue’s study, who used multi antigens containing 2 or 3 genes of GRA2, SAG1, and ROP_2_ (29). The same was fund by Vercommen, using ROP2, GRA1, and GRA7 (14) and by Dautu *et al.* using plasmids containing bradyzoite antigen-1 (BAG1), apical membrane antigen 1 (AMA1), microneme protein-2 associated protein (M2AP) and MIC2 (27). The present study revealed that the first phase of pcMIC3’s vaccination elicited quick humoral responses and become more effective over time upon further vaccination. Xue and co-workers challenged two groups of mice with either of the two plasmids pSAG-ROP2-GRA2 and pSAG-ROP2 and reported that the former vaccine produced higher amount of IgG_2a_ and therefore stronger Th1 response (29). Moreover, Desolme *et al.* stated that both pcGRA4 and pcGRA4/pGM-CSF plasmids elicited increased production of IgG in mice with no significant differences in their efficacy (28). Taking into account, it seems that application of MIC3 and pCAGGS-IL12 conjugate can result in increased immunity responses that are related to Th1. Studies showed that genetic adjuvants increase antibody production, so that cytokines containing plasmids modulate immune responses towards actuation and development of Th1 (IFN-γ), whereas using recombinant proteins of antigens as adjuvant actuates Th2 (IL-4) type. Yang *et al*. found that recombinant TgMIC3 has a cross protective effect against both *T. gondii* and *Neospora caninum *(32). 

In recent studies, we showed the importance of MIC antigens in the progress and development of vaccines (33, 34). In a study, Ismael *et al.* showed that a DNA vaccine containing MIC3 significantly increased cellular immunity responses and secretion of IFN-γ and IL-2 cytokines in immunized mice. They also used a plasmid that encoded granulocyte-macrophage colony-stimulating factor (GM-CSF) as adjuvant (35-38). In the present study, we observed increase in cellular immune responses as a result of designed DNA vaccine, especially when we used IL-12 as a genetic adjuvant. 

## Conclusion

The results of the present study showed that immunity responses, specifically the cell immunity were constructed in mice after immunization with pc-MIC3 (in combination with IL-12 as a genetic adjuvant). However, this could not provide complete protection to mice against the virulent strain of *T. gondii*. 
